# Expression of Toll-Like Receptor -7 and -9 in B Cell Subsets from Patients with Primary Sjögren’s Syndrome

**DOI:** 10.1371/journal.pone.0120383

**Published:** 2015-03-19

**Authors:** Marie Karlsen, Torbjørn Hansen, Hilde H. Nordal, Johan G. Brun, Roland Jonsson, Silke Appel

**Affiliations:** 1 Broegelmann Research Laboratory, Department of Clinical Science, University of Bergen, Bergen, Norway; 2 Department of Clinical Science, University of Bergen, Bergen, Norway; 3 Department of Immunology and Transfusion Medicine, Haukeland University Hospital, Bergen, Norway; 4 Department of Rheumatology, Haukeland University Hospital, Bergen, Norway; Institute of Immunology, Rikshospitalet, NORWAY

## Abstract

**Introduction:**

Sjögren’s syndrome (SS) is a rheumatic autoimmune disease characterized by inflammation of exocrine glands. As autoantibodies are present in a majority of patients, B cells have been suggested to play an important role in onset and development of the disease. Toll-like receptors (TLRs) are pattern recognition receptors triggering innate immune responses. Since an increased expression of TLRs has been detected in other rheumatic diseases the purpose of this study was to explore TLRs in B cells of SS patients.

**Methods:**

The expression of TLR-7 and -9 in B cell subsets of 25 patients with primary SS (pSS) and 25 healthy controls was analysed in peripheral blood using flow cytometry and real time quantitative PCR.

**Results:**

We detected similar levels of CD19^+^ B cells in pSS patients and healthy controls. An increased number of naïve B cells, as well as fewer pre-switched memory B cells were found in pSS patients. No significant differences were observed in TLR-7 and -9 expression in B cells between pSS patients and healthy controls.

**Conclusion:**

This study shows that pSS patients have an alteration in the B cell subpopulation composition compared to controls, with less pre-switched memory B cells and more naïve B cells. We did not detect any significant disparities in TLR-7 and -9 expression between the two groups.

## INTRODUCTION

Primary Sjögren’s syndrome (pSS) is a chronic, inflammatory autoimmune disease characterized by destruction of the salivary and lacrimal glands leading to dryness of the mouth (xerostomia) and the eyes (keratoconjunctivitis sicca) [1]. Since these symptoms occur after the onset of disease, many patients already have severe destruction in their exocrine glands when diagnosed. These damages cannot be reversed, and may cause reduction in quality of life for the patients [1]. The lack of a single symptom impedes the diagnosis of Sjögren’s syndrome [2] [3], and a diagnostic delay of 3–11 years has been reported [4]. A recent study revealed that autoantibodies are present years before clinical symptoms arise[5]. If this disease was detected before symptom onset, destruction in exocrine glands might be reduced, and quality of life could be maintained. The disease affects mainly women above 40 years of age, and has a prevalence of around 0,05% [6]. Hallmarks of pSS are mononuclear cell infiltrates within the glandular tissue and presence of autoantibodies against SSA/Ro and SSB/La antigens [1]. Because of the production of autoantibodies, B cells have been recognized as important cells in the pathogenesis of Sjögren’s syndrome [7].

There is no cure, and only symptomatic treatment can be offered to these patients. In the recent years, several trials using B cell depletion with Rituximab (anti CD20) have been performed with variable results [8–11]. While some studies report dampening of disease activity [8,11], others did not detect similar results [9,10]. A limiting factor may be less effective B cell depletion in tissues compared to blood [12]. However, the majority of these trials report improvement in fatigue after treatment with rituximab. The challenge remains to verify that these improvements are caused by B cell depletion, as all patients were treated with methylprednisolone in these trials.

To generate new and more effective treatments for these patients, more information on the cause and the mechanisms behind pSS is needed.

Toll-like receptors (TLRs) are pattern recognition receptors (PRRs), sensing conserved pathogen-associated molecular patterns (PAMPs). They function as sentinels, alerting the immune system of threatening microbial invasion [13,14]. TLRs localized on the cell surface bind molecular components exposed on microbes, while TLR-3, -7, -8 and -9 are located in endosomes and bind microbial nucleic acids. PAMP binding triggers signalling via the adaptor proteins MyD88 or TRIF, leading to activation of transcription factors NF-κB and AP-1, resulting in secretion of cytokines and up-regulation of co-stimulatory molecules.

Moreover, TLRs of B cells seem to participate in activation of B cells. For naïve B cells, it has been reported that three signals are required for optimal activation: 1) antigen binding to the B cell receptor, 2) specific binding of a T cell receptor, and 3) engagement of a TLR [15].

Autologous nucleic acids might in some situations get access to and bind endosomal TLRs in a B cell, and thereby stimulate the cell. One way this may happen is that B cells with BCR specific for nucleic acids or proteins in complex with nucleic acids internalize the antigens by receptor-assisted endocytosis. This would make the nucleic acids available for binding to endosome-associated TLR. Thus, these antigens may be both autoantigens and autoadjuvants [16,17]. It is an open question whether some disease-associated antibodies are elicited in this way; however, inhibition of TLR-9 blocked antibody production by B-cells from systemic lupus erythematosus (SLE) patients, and in one murine SLE model, B cell overexpression of TLR-7 resulted in increased production of autoantibodies to RNA/protein complexes and worsening of disease [18,19].

Little is known about the role of TLR expression of B cells in patients with Sjögren’s syndrome. Since an increased expression of TLRs have been detected in other rheumatic diseases, the aim of this study was to investigate the expression levels of TLR-7 and -9 in peripheral blood B cell populations of patients with pSS compared to healthy controls. Our results showed that pSS patients have an increased number of naïve B cells, and fewer pre-switched memory B cells, compared to healthy controls. However, there was no difference in TLR-7 or -9 expression between pSS patients and controls.

## METHODS

### Blood samples

25 pSS patients and 25 healthy controls were included. The patients were recruited from the Department of Rheumatology at the Haukeland University Hospital in Bergen, Norway. All pSS patients included in the study have been diagnosed according to the American-European consensus group criteria (AECC) [2]. Clinical, laboratory and histopathological characteristics of the patient group are shown in Table 1. The control group consisted of gender- and age-matched healthy blood donors from the Bloodbank, Department of Immunology and Transfusion Medicine, at the Haukeland University Hospital, Bergen, Norway. All studied subjects gave their informed consent and the Regional Committee for Research Ethics approved the study (#242.06).

**Table 1:** Clinical Characteristics of the individuals included in this study.

Cohort characteristics		
	Sjögren’s syndrome	Healthy controls
Number	n = 25	n = 25
Age in years (mean)	59±14	57±5
Range	19–77	44–66
Female:male	25:0	25:0
Clinical features		
Anti-Ro+ (Anti-La÷)	7	
Anti-La+ (Anti-Ro÷)	0	
Both anti-Ro and anti-La+	10	
Both anti-Ro and anti-La+	8	
ANA	20	
Positive saliva test*	13	
Positive Schirmer’s test**	16	
Extraglandular manifestations	12	
Focus score***		
Positive	15	
Negative	4	
Not analysed	6	
Medication		
No medication	12	
Hydroxychloroquine	5	
Prednisolone	3	
Hydroxychloroquine and prednisolone	4	
Methotrexate	1	

*Positive saliva test: Unstimulated whole saliva <1,5ml/15min

**Positive Schirmer’s test: Wetting of tears flow test strip ≤5mm/5min

***Positive focus score: ≥1 focus (≥50 cells) per 4mm^2^

### Isolation of cells from whole blood

Heparinized whole blood (25–35 ml) was collected from pSS patients and healthy controls. PBMC were isolated by discontinuous gradient centrifugation using Ficoll-Paque/Lymphoprep (Axis shield PoC AS, Oslo, Norway), and washed 3 times with PBS+0.1% BSA. The isolated PBMC were stained directly for flow cytometry.

### Immunostaining

Surface marker staining was performed for 10 min at room temperature (RT), while intracellular staining was done for 30 min at 4°C. To inhibit unspecific binding, FcR Blocking Reagent (5μl/10^6^ cells; Miltenyi Biotec Norden AB, Lund, Sweden) was used before both surface and intracellular staining. PBMC (1x10^6^) were stained for surface markers using anti-CD19-PE-Cy7 (SJ25C1), anti-CD25-Alexa Fluor 700 (M-A251), anti-CD27-PerCP-Cy5.5 (M-T271), anti-CD45-APC-H7 (2D1) and anti-IgD-FITC (IA6-2) (all from BD Biosciences, Heidelberg, Germany). These cells were then washed twice with FACS buffer (PBS+0.5% BSA) before they were fixed according to the manufacturer’s protocol (Cytofix/Cytoperm, BD Biosciences). Next the intracellular targets anti-TLR7-PE (533707)(R&D systems, Minneapolis, MN, USA) and anti-TLR9-Alexa Fluor 647 (26C593.2)(Imgenex, San Diego, CA, USA) were stained for 30 min at 4°C, followed by 2 washes with perm/wash buffer (BD Biosciences) before the cells were resuspended in FACS buffer and analyzed on a BD LSR Fortessa. Fluorescence Minus One (FMO) controls were used to set the gates. FlowJo software (Tree Star, Ashland, OR) was used for analyses.

### RNA isolation, cDNA synthesis and quantitative real time PCR

Untouched B cells were isolated from PBMC by negative selection, using the B cell isolation kit II (Miltenyi Biotec Norden AB, Lund, Sweden). The purity of the isolated cells was analyzed by flow cytometry using a mouse anti-human CD19 antibody (SJ25C1). On average, 86.3% (pSS patients) and 91.0% (controls) of the cells were CD19^+^.Total RNA was isolated from 1x10^5^ B cells using RNeasy Mini Kit (QIAGEN, Hilden, Germany) according to the manufacturer’s protocol. RNA concentrations were measured using a Nanodrop Spectrophotometer NS-1000 (Thermo Scientific, Wilmington, DE, US), and cDNA was synthesized from 16 ng of RNA using High-Capacity RNA-to-cDNA Kit (Applied Biosystems, Carlsbad, USA) according to the manufacturer’s recommendations. Taqman Gene Expression Assays (TLR7; Hs00152971_m1, TLR9; Hs00152973_m1, Applied Biosystems) were used to analyze TLR-7, and -9 transcription levels in a quantitative real time PCR, using an ABI 7500 real time PCR system (Applied Biosystems). Samples were run in duplicates and GAPDH (Hs02758991_g1, Applied Biosystems) was used for normalization (S1 Fig.).

### Statistical analysis

Median is indicated in all figures. Statistical analyses were performed using Mann-Whitney test or 1 way ANOVA. Significance was set at P< 0.05. All statistical calculations were performed with Prism 5 (GraphPad Software, Inc., USA).

## RESULTS

### Similar number of CD19^+^ B cells in pSS patients and healthy controls, but altered CD27 expression

**Fig 1:**
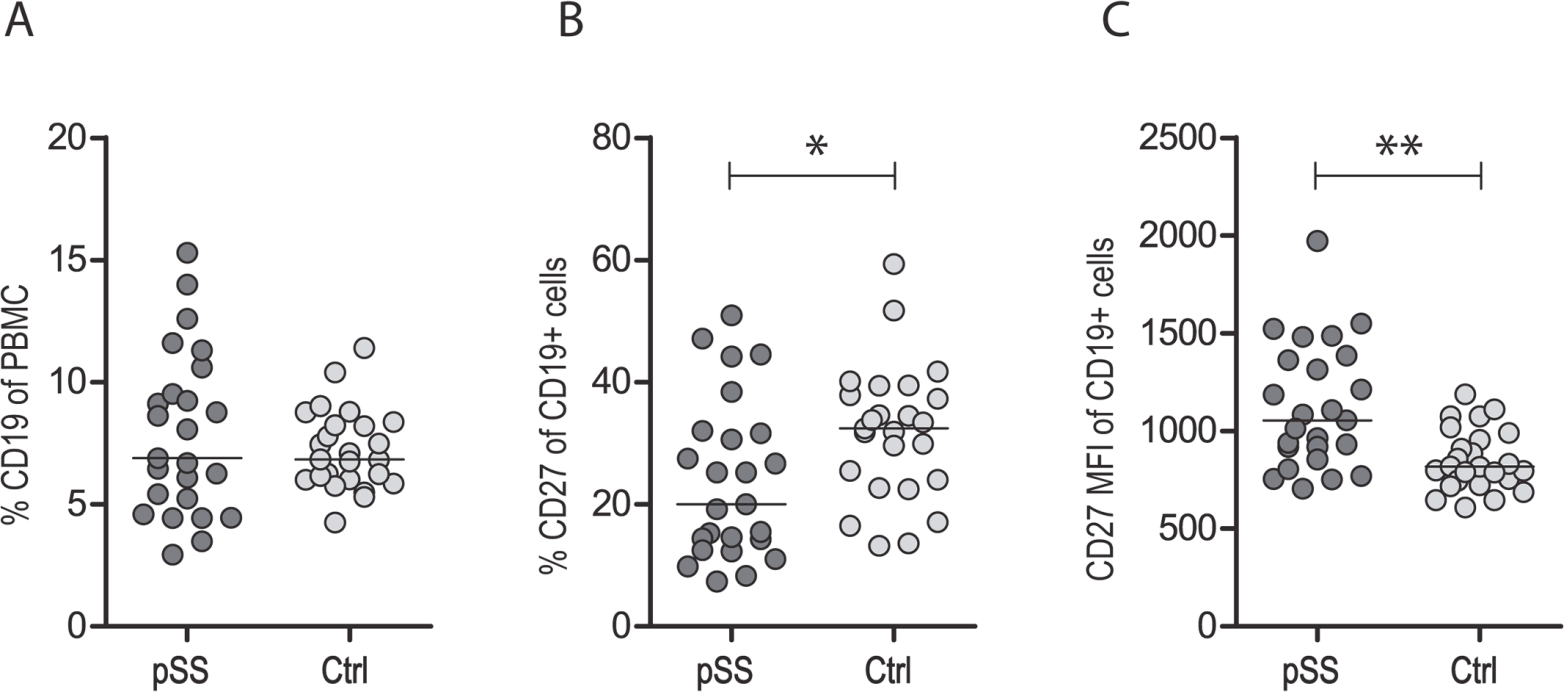
Similar numbers of CD19^+^ B cells in pSS patients and controls, but decreased amounts of CD27^+^ cells. No significant difference was identified in number of CD19^+^ B cells in PBMC comparing pSS patients and healthy controls, although a greater variation was detected in the patient group (A). Fewer CD19^+^ B cells expressed CD27 in pSS patients compared to healthy controls, yet the MFI values were significantly higher in pSS (C). The bar indicates the median; *: p <0.05, **: p <0.01.

**Fig 2:**
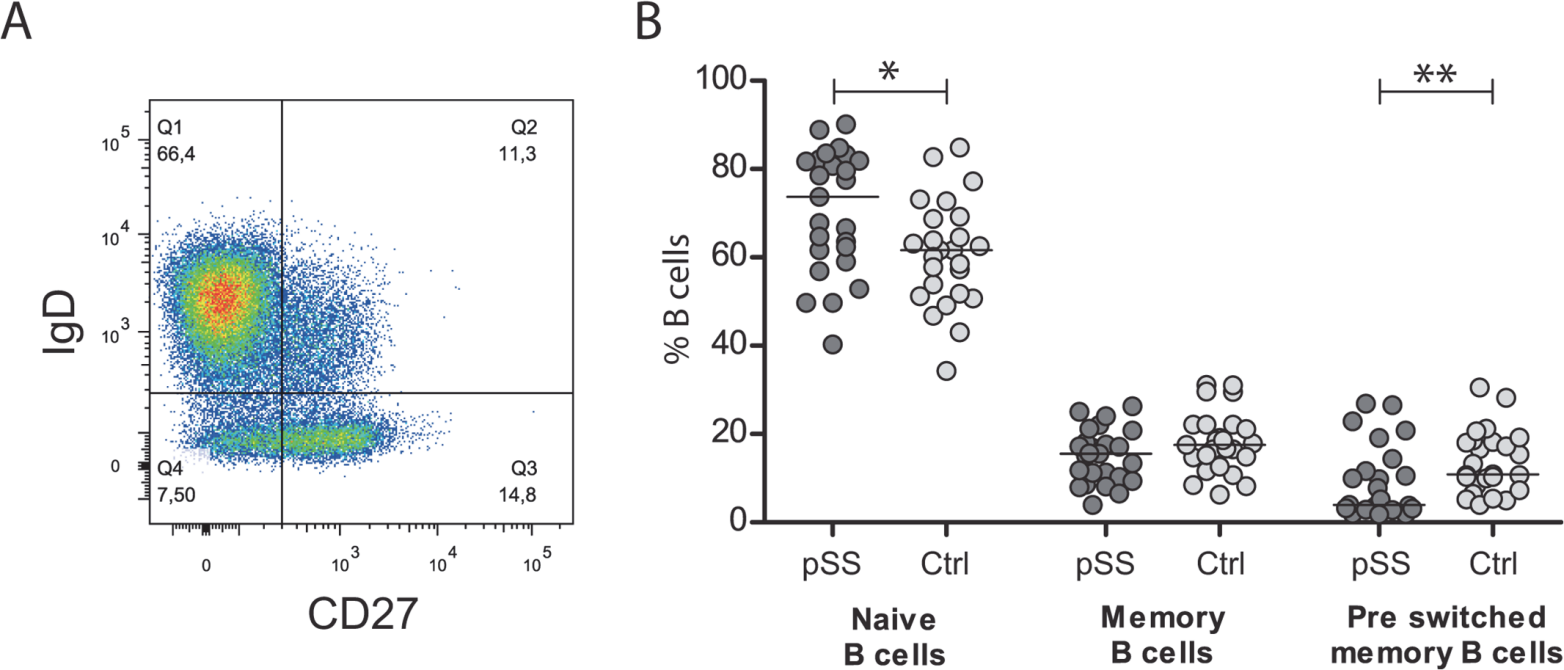
Altered distribution of B cells between subsets in pSS compared to healthy controls. A) B cells in PBMC were detected by gating CD19^+^ CD45^+^ cells. B cell subsets were defined by CD27 and IgD expression. Naïve B cells were defined as CD19^+^CD27^−^IgD^+^ (Q1), memory B cells as CD19^+^CD27^+^IgD^−^ (Q3), and pre-switched memory B cells as CD19^+^CD27^+^IgD^+^ (Q2). B) An increased number of naïve B cells was detected in pSS patients compared to healthy controls. No differences were found in amount of memory B cells between the groups, while a decreased level of pre-switched memory B cells were discovered in pSS patients. The bar indicates the median; *: p <0.05, **: p <0.01.

The number of CD19^+^ cells expressing CD27 and IgD were analysed for pSS patients and controls to differentiate between naïve B cells (CD19^+^CD27^−^IgD^+^), pre-switch memory B cells (CD19^+^CD27^+^IgD^+^) and memory B cells (CD19^+^CD27^+^IgD^−^). Total numbers of CD19^+^ cells in PBMC were similar between pSS patients and controls (Fig. 1A), but there were more variations in the pSS group than the controls (pSS: 2.9–15.3% CD19^+^ cells, controls: 4.3–11.4% CD19^+^ cells). Patients with the lowest and highest numbers of B cells did not have any extraglandular manifestations that could have a correlation with the number of B cells, such as leukopenia or hypergammaglobulinemia. However, we did notice that 5 of the 6 patients with less than 5% CD19+ B cells within the PBMC were SSB/La negative. Since many published studies on B cell subsets in pSS and other autoimmune diseases define memory B cells as CD19^+^CD27^+^, we also looked at this subset. When analysing the number of CD19^+^ cells expressing CD27, we found a significantly reduced number in pSS patients. Interestingly, even though the patients had fewer CD27^+^ B cells, they still had higher median fluorescence intensity (MFI) values for CD27 (Fig. 1B). We detected a significantly higher number of naïve B cells (CD19^+^CD27^−^IgD^+^) in pSS patients, similar amounts of memory B cells (CD19^+^CD27^+^IgD^−^), and a reduced number of pre-switched memory B cells (CD19^+^CD27^+^IgD^+^) compared to healthy controls (Fig. 2).

### TLR expression in B cell subsets

**Fig 3:**
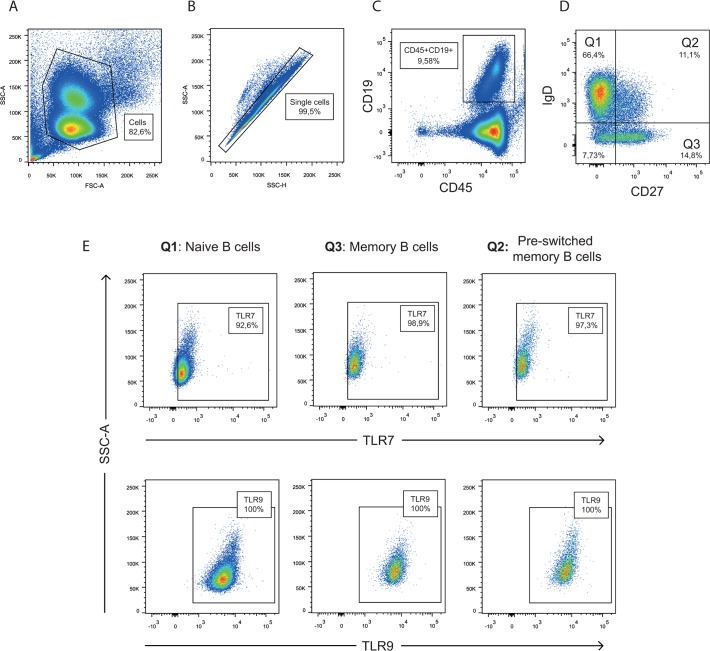
Gating strategy for the detection of TLR-7 and -9 in B cell subsets. PBMC were isolated from whole blood and stained for surface markers before cells were fixed, permeabilised and stained for TLR-7 and -9. FSC and SSC were first used to gate out debris (A) and SSC-A and SSC-H was utilized to eliminate duplicates (B). Further gating was done on CD45 and CD19, to target B cells (C). To separate between the different B cell populations, we gated on CD27 and IgD (D), followed by TLR-7 and -9 expression on these subsets (E). Data from one representative patient is shown.

**Fig 4:**
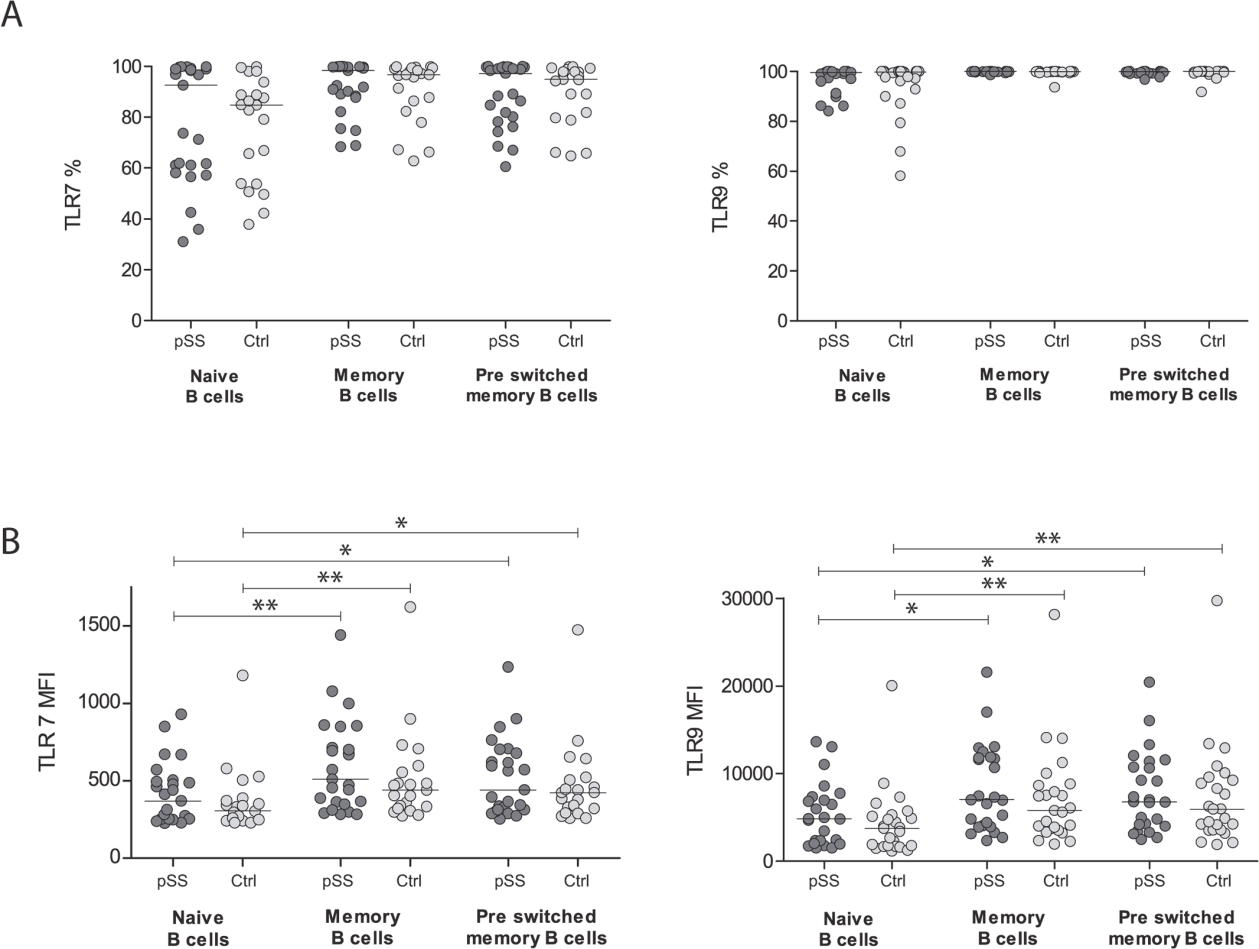
No significant changes in TLR-7 and -9 expression between pSS patients and controls. No significant variations were found in TLR-7 and -9 expression between pSS patients and controls. Memory and pre-switched memory B cells had an upregulation of TLR-7 and -9 MFI values, compared to naïve B cells in both patients and controls. Percent positive cells are shown in A, and MFI values are seen in B. The bar indicates the median and *: p <0.05, **: p <0.01.

**Fig 5:**
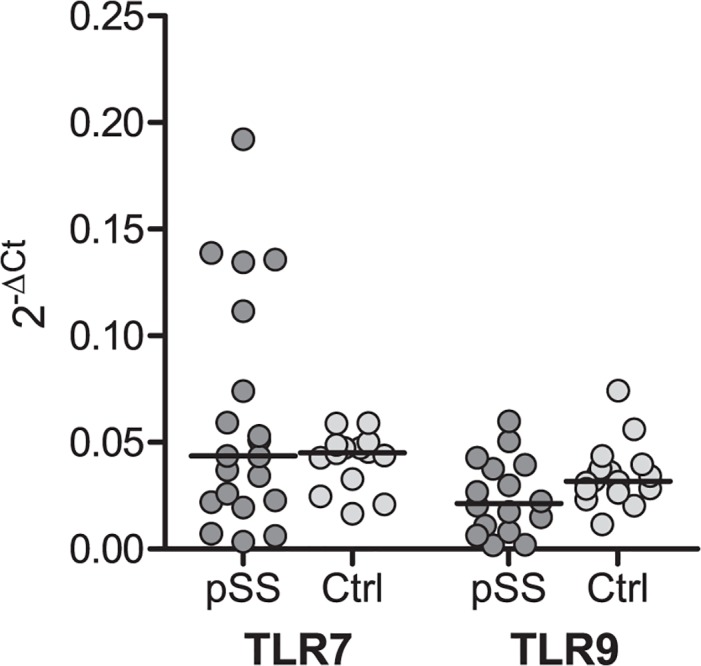
Similar TLR-7 and -9 mRNA level in pSS patients and controls. No significant disparities were detected in mRNA level, however a slightly lower level of TLR-9 was noticed in pSS. RNA was isolated from negatively isolated B cells, and cDNA was synthesized with High-Capacity RNA-to-cDNA Kit. Taqman gene expression assays were used to detect TLR-7 and -9. GAPDH was used for normalization. Median is indicated in the figure.

In order to analyse TLR expression in B cell subsets, PBMC were stained with surface markers to identify different B cell populations before cells were fixed, permeabilized and stained for TLR-7 and -9. We used CD27 and IgD to distinguish the B cell subsets, and the gating strategy is shown in Fig. 3. No significant differences in TLR-7 and -9 expression were detected between pSS patients and controls (Fig. 4), though different expression levels of the TLRs were observed in the various B cells subsets. Naïve B cells expressed less TLR-7 and -9 than the memory B cell subsets in both, patients and controls (Fig. 4B). To explore if there were differences between protein expression and amount of transcripts, we analysed the mRNA levels of TLR-7 and -9 in isolated B cells from most of the pSS patients and healthy controls (n = 21 and 18, respectively). No significant changes were detected (Fig. 5).

### Effects of medication

**Fig 6:**
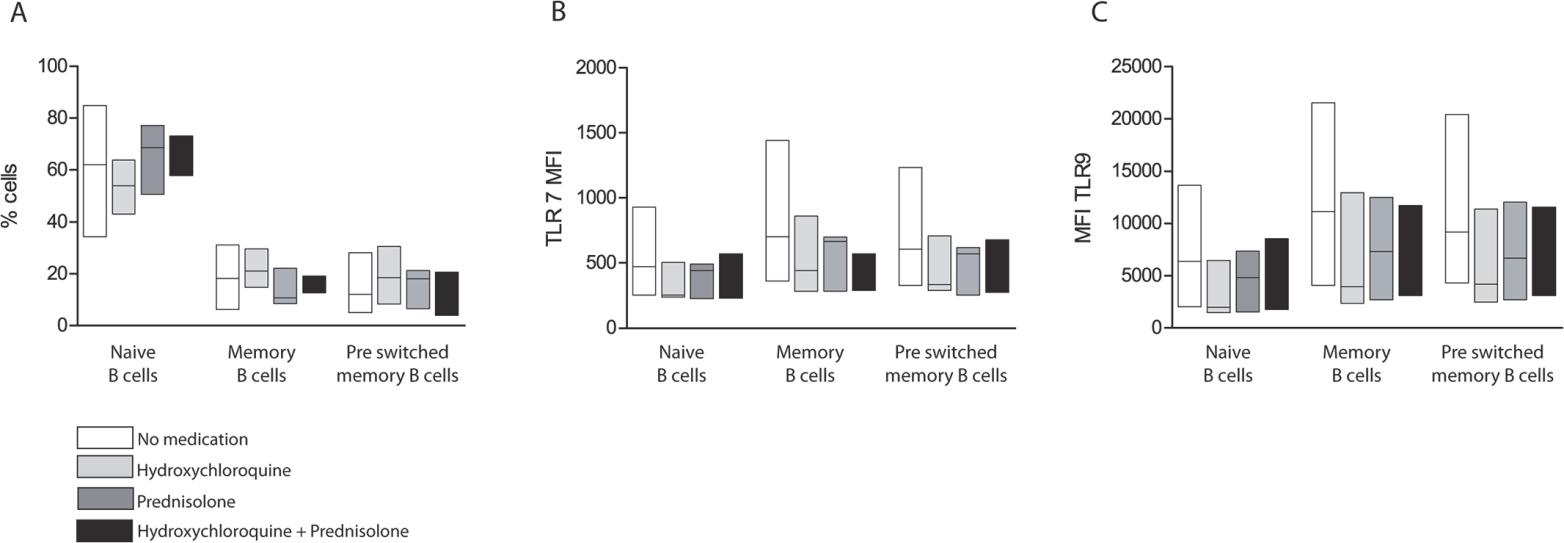
The medications used did not have a significant effect on B cell subsets or TLR expression. No significant differences were detected in the distribution B cells between the different subsets (A), or in TLR-7 and -9 expression (B and C). Patients not receiving any medications had a slightly increased TLR-7 and -9 expression in all B cell subpopulations, compared to those on any medication (B and C). The median is indicated as a line in the bars. No medication; n = 12, hydroxychloroquine; n = 5, predisolone; n = 3, and hydroxychloroquine+prednisolone; n = 4.

Since many pSS patients take medications to relieve symptoms, we analysed if the medication had an impact on our results. In our patient group, 12 did not take any medication, 5 patients were on hydroxychloroquine, 3 on prednisolone and 4 patients on both hydroxychloroquine and prednisolone. Only one patient received methotrexate and was therefore not included in the analysis. No significant differences were found between the four groups in the amount of naïve B cells, memory B cells or pre-switched memory B cells (Fig. 6A), nor the expression of TLR-7 and -9. However, a weak trend indicated highest expressions of TLR-7 and -9 in the patients that were not medicated (Fig. 6B and 6C).

## DISCUSSION

We used CD19 as B cell marker due to its expression on all developmental stages of B cells [20]. To identify B cell subsets, we utilized CD27 and IgD on B cells. Some of the previous studies on B cells in pSS used CD27 alone to separate between naïve and memory B cells [21,22]. These studies showed that pSS patients have elevated numbers of CD27^−^ B cells and reduced numbers of CD27^+^ B cells than healthy controls [21,22]. This is in accordance with our data (Fig. 1). We found that pSS patients had more naïve B cells than healthy controls. However, when defining memory cells as CD27^+^/ IgD^−^, and naïve B cells as CD27^−^/IgD^+^ cells, some CD27^−^/IgD^+^/CD38^high^ B cells could be included in the naïve B cell subset. This subset amounts to 2.4–5.4% of B cells in a healthy individual [23]. However, these cells are trastitional B cells that eventually develop into naïve B cells. We found that pSS patients had more naïve B cells than healthy controls[23]. Patients with pSS had significantly less pre-switched memory B cells than controls (Fig. 2), which is in line with previous studies with pSS patients [22], as well as in early RA patients [24].

In this study we analysed TLR-7 and -9 expression in different B cell subsets of pSS patients. Since an upregulation of TLR-7 and -9 has been reported earlier using PBMC from pSS patients [25], and both PBMC and B cells from SLE patients [26–28], we analysed TLR-7 and -9 expression in various B cell populations from pSS patients. Several hallmarks of pSS indicate involvement of B cells, and upregulations of TLRs on B cells have been detected in other rheumatic diseases. We did not detect increased TLR-7 or -9 expression in pSS patients in any of the B cell subsets analysed (Fig. 4), even though these TLRs have been shown to be increased in PBMC in pSS patients. An explanation for the similar levels of TLR-7 and -9 in B cells might be that we analysed pure B cells, while PBMC are a heterogeneous cell population, and TLRs could be upregulated in other cells than B cells. TLR9 has been shown to be expressed at similar levels in B cells, T cells and monocytes [29].Although we did not find any upregulations, it does not necessarily mean that TLR-7 and -9 are not involved in the pathogenesis of pSS. TLR signalling can be affected without higher expression of the receptors. One example is that excess type I IFNs have shown to induce stronger TLR-7 responses by naive B cells [30], and type I IFNs, transcription factors induced by IFN, and pathways related to IFN signalling have been found overexpressed in pSS [31,32].

Our data obtained by flow cytometry show that all B cells subsets express TLR-7 and -9, with lower levels in naïve B cells. A previous study using mRNA showed that TLR-7 and -9 are expressed at high levels in memory B cells, while only low to undetectable levels were found in naïve B cells [33]. Increased level of TLRs detected on memory B cells could be a result of the activation these cells have been through. When naïve B cells encounter and bind antigens to their B cell receptor, they are activated develop into plasma or memory B cells. During this activation, an upregulation of TLRs might take place.

In PBMC from both pSS and SLE patients, increased TLR-7 and -9 expression were detected [25,26], but these studies were also performed on mRNA level only, which does not necessarily correspond to amount of protein [34,35]. In SLE patients, elevated levels of TLR-2, -3, -4, -5, -6, -8 and -9 have been detected in monocytes, T cells and B cells by flow cytometry. [27]. Flow cytometry is a good and well known method for detecting actual protein level present in or on cells, however all methods have some limitations or pitfalls. The use of controls is important in flow cytometry to obtain reliable results. In this study we have used unstained cells as negative control, and we have used fluorescence minus one (FMO) to remove spill over signals from the other detectors. Some consider isotype controls to be a necessary control to eliminate nonspecific staining, while others find FcR blocking before staining to be an adequate control to reduce the potential unspecific binding. In addition it should also be mentioned that the different antibody isotypes have different affinities for binding Fc receptors, meaning that some antibodies might give more unspecific binding that others [36]. In our study we detected the same result at both mRNA and protein level, which is a good verification of the obtained result.

Interestingly, besides endosomal expression, also surface expression of TLR-9 on PBMC and B cells has been detected in several studies during the last decade. Upon lipopolysaccharide (LPS) stimulation, surface expression of TLR-9 on PBMC increased four-fold [37], and stimulation with interleukin (IL)-2, IL-10, CD40L in absence or presence of TLR-9 ligand ODN 2006 was shown to increase surface TLR-9 as well as IL-6 produced by B cells, suggesting that TLR-9 engagement is followed by continued IL-6 production [38]. Analyses indicated that surface TLR-9 on B cells act differently than endosomal TLR-9 as these surface receptors do not bind CpG. And surprisingly, when activated with anti-TLR-9 antibody, CD25 expression was not upregulated on B cells. Surface TLR-9 stimulation did not induce proliferation as activation with CpG in B cells. In fact, it was detected that activation after CpG stimulation was inhibited by the presence of anti-TLR9 antibody, as downregulation of CD25 was observed in addition to a dampening of the proliferative response [39]. This suggests that surface TLR-9 might be a negative regulator of the endosomal TLR-9 induced B cell response. We could not differentiate between surface and endosomal TLR-9 in our study. However, it is not likely that there would exist a difference in surface TLR-9 between pSS patients and controls since we did not detect a difference in total TLR-9 expression. Therefore, there would have to be a corresponding up or down regulation in endosomal TLR-9 expression.

We used CD19 as B cell marker since it is expressed on all developmental stages of B cells [20]. To identify B cell subsets, we utilized CD27 and IgD on B cells. Some of the previous studies on B cells in pSS used CD27 alone to separate between naïve and memory B cells [21,22]. These studies showed that pSS patients have elevated numbers of CD27^−^ B cells and reduced numbers of CD27^+^ B cells than healthy controls [21,22]. This is in accordance with our data (Fig. 1). When defining naïve B cells as CD27^−^/IgD^+^ cells, and memory cells as CD27^+^/ IgD^−^, we found that pSS patients had more naïve B cells than healthy controls. Patients with pSS had significantly less pre-switched memory B cells than controls (Fig. 2), which is in line with previous studies with pSS patients [22], as well as in early RA patients [24].

## CONCLUSION

In conclusion, our study shows that pSS patients have an increased number of naïve B cells in peripheral blood, as well as fewer pre-switched memory B cells compared to healthy controls. However we did not find any significant disparities in expression of TLR-7 and -9 between pSS patients and healthy controls.

## Supporting Information

S1 Fig TLR-7 and -9 mRNA normalized to GAPDH. RNA was isolated from negatively isolated B cells, and cDNA was synthesized with High-Capacity RNA-to-cDNA Kit. Taqman gene expression assays were used to detect TLR-7 and -9. ΔCt values of TLR-7 and -9 normalized to GAPDH are shown, median is indicated in the figure.
(EPS)
